# *Candida parapsilosis* candidemia in children admitted to a tertiary hospital in Turkey: clinical features and antifungal susceptibility

**DOI:** 10.1128/spectrum.00564-24

**Published:** 2024-06-12

**Authors:** Fatma Tuğba Çetin, Ümmühan Çay, Murat Polat, Fatma Kılınç, Asena Ünal, Nisa Nur Tapaç, Özlem Özgür Gündeşlioğlu, Derya Alabaz, Sevcan Bilen, Filiz Kibar, Nazlı Totik

**Affiliations:** 1 Department of Pediatric Infection, Faculty of Medicine, Cukurova University, Adana, Turkey; 2 Department of Child Health and Diseases, Faculty of Medicine, Cukurova University, Adana, Turkey; 3 Department of Pediatric Emergency Medicine, Cukurova University, Adana, Turkey; 4 Department of Microbiology, Faculty of Medicine, Cukurova University, Adana, Turkey; 5 Department of Biostatistics, Faculty of Medicine, Cukurova University, Adana, Turkey; University of Wisconsin—Madison, Madison, Wisconsin, USA

**Keywords:** antifungal sensitivity, *Candida parapsilosis*, child, pediatric

## Abstract

In recent years, the incidence and drug resistance of *Candida parapsilosis* have increased. Our study aimed to determine the antifungal sensitivity of *C. parapsilosis* and the clinical and demographic characteristics of children with candidemia. Two hundred pediatric patients with *C. parapsilosis* candidemia were included in the study between 1 January 2010 and 1 August 2023. Clinical samples were evaluated on a BACTEC-FX-40 automatic blood culture device (Becton Dickinson, USA). Yeast isolates were identified to the species level via identification cards (YST) using the VITEK 2 Compact (bioMeriéux, France) system. Antifungal susceptibility was performed using antifungal cell cards (AST-YST01). Approval for the study was received from the “University Faculty of Medicine” Hospital Clinical Research Ethics Committee. Non-catheter candidemia was detected in 127 (63.5%) patients, and catheter-related candidemia was detected in 73 (36.5%) patients. It was observed that the patients’ history of malignancy, mechanical ventilation, urinary catheter, nasogastric tube, and intensive care unit stay was associated with *C. parapsilosis* mortality. The mortality rate from candidemia was 9.5%. The most frequently preferred antifungal agents were amphotericin B and fluconazole. The fluconazole drug resistance rate was found to be 6%, and the amphotericin B drug resistance rate was 4%. Because *C. parapsilosis* candidemia mortality rates can be high depending on risk factors and clinical characteristics, it is important to initiate appropriate and timely antifungal therapy. We think that our study can provide important information about the clinical profiles, distributions, susceptibility profiles, and control of antifungal resistance of *C. parapsilosis* isolates.

**IMPORTANCE:**

It has been observed that the frequency and antifungal resistance of *Candida parapsilosis* have increased recently. In our study, we aimed to determine the antifungal sensitivity of *C. parapsilosis* and the clinical and demographic characteristics of children with candidemia. It was observed that the patients’ history of malignancy, mechanical ventilation, urinary catheter, nasogastric tube, and intensive care stay was associated with *C. parapsilosis* mortality. The mortality rate from candidemia was 9.5%. The most frequently preferred antifungal agents were amphotericin B and fluconazole. The fluconazole drug resistance rate was found to be 6%, and the amphotericin B drug resistance rate was 4%. Because *C. parapsilosis* candidemia mortality rates can be high depending on risk factors and clinical characteristics, it is important to initiate appropriate and timely antifungal therapy.

## INTRODUCTION

The incidence of invasive fungal infections is increasing worldwide, and these infections are associated with high morbidity and mortality. *Candida* species are the most common cause of invasive fungal infections (1).

More than 90% of invasive candidiasis is caused by *Candida albicans*, *Candida parapsilosis*, *Candida glabrata*, *Candida tropicalis*, and *Candida krusei*. The epidemiology of invasive candidiasis has been changing in recent years, and the most common non-albicans strain is *C. parapsilosis* (2, 3). *C. parapsilosis* is frequently isolated from patients with malignancies, immunocompromised patients, and patients hospitalized for long periods in the intensive care unit (4). Additionally, the frequency of non*-Candida albicans* species causing nosocomial infections has increased in the last few decades (5).

Conditions such as multiple antibiotic therapy, total parenteral nutrition (TPN), nasogastric tube, mechanical ventilation, admission to intensive care, and neutropenia are risk factors for candidemia (1). Since mortality rates in candidemia (40%–60%) may be high depending on risk factors and clinical characteristics, it is important to initiate appropriate and timely antifungal treatment (6). For effective antifungal treatment, it is important to correctly identify the candidemia agent and know its sensitivity to antifungal agents (6, 7).

There are very few studies and information regarding antifungal drug resistance in *C. parapsilosis*. In particular, the increasing use of antifungal agents may lead to resistant *Candida* species (8). Antifungal susceptibility results of *C. parapsilosis* strains may affect treatment choices and, as a result, morbidity and mortality rates may change.

This study aimed to determine the antifungal sensitivity of *C. parapsilosis* and the clinical and demographic characteristics of pediatric patients with candidemia.

## MATERIALS AND METHODS

This is a retrospective study conducted in a single-center tertiary hospital. Pediatric patients whose blood and catheter cultures showed *C. parapsilosis* growth and whose signs and symptoms were compatible with candidemia between 1 January 2010 and 1 August 2023 were included in our study. Patients older than 18 years and patients with insufficient data were excluded.

If the patient with a bloodstream infection and catheter does not have another focus of infection, the same microorganism is produced from peripheral blood culture and semiquantitative (>15 cfu) or quantitative (>100 cfu) culture of the catheter tip or in simultaneous quantitative blood cultures. A catheter-related infection was defined as a growth rate of ≥3/1 in the central venous catheter/peripheral blood culture or detection of growth >2 hours earlier in the blood culture taken from the central venous catheter than in the simultaneously taken peripheral blood culture. Candidemia cases that did not meet this criterion were defined as non-catheter-associated candidemia (9, 10). In patients with previous candidemia, *Candida* overgrowth 30 days after the first negative culture was considered a new episode of candidemia. Culture and antibiogram data regarding candidemia were obtained retrospectively from microbiology laboratory records. Demographic and clinical characteristics, underlying disease, risk factors, antifungal susceptibility test results, treatment, and prognosis of infections caused by *C. parapsilosis* were obtained from patient files.

The time from hospital admission to the growth of *Candida* species in the blood culture was recorded. Mortality within 30 days after *Candida* overgrowth was defined as the overall mortality and mortality rate due to candida. The overall mortality rate was calculated as the number of patients who died/all patients, regardless of the cause of death. The candidemia-related mortality rate was calculated as the percentage of patients who died from candidemia/all patients (11). Accordingly, 7-day and 30-day mortality rates were calculated.

Organ scanning was performed on all patients with abdominal ultrasonography, abdominal tomography if necessary, eye examination, and echocardiography.

Clinical samples sent to the microbiology laboratory with appropriate blood culture bottles were incubated in the BACTEC-FX-40 automatic blood culture device (Becton Dickinson, USA) and monitored for 7 days. Growth signals were seen in the smear preparations taken from the bottles, and Gram staining was performed. In addition, these samples were passed through sheep blood agar, MacConkey agar, and Sabouraud dextrose agar. Colony morphology was evaluated after 18–24 hours incubation at 37°C. Isolates compatible with yeast morphology were identified to species level using identification cards (YST) with the VITEK 2 Compact (bioMeriéux, France) system. Due to laboratory conditions, *C. parapsilosis* complex subspecies could not be identified. *C. parapsilosis* refers to all strains of the *C. parapsilosis* complex. At the same time, amphotericin B, caspofungin, micafungin, fluconazole, flucytosine, and voriconazole sensitivities were evaluated using antifungal sensitivity cards (AST-YST01). Minimum inhibitory concentration (MIC) limit values were determined in the antibiotic sensitivity test. *C. parapsilosis* MIC breakpoint was evaluated according to the CLSI M60 2017 guideline (12). MIC ≥ 8 mg/L for fluconazole, MIC ≥ 8 mg/L for caspofungin, MIC ≥ 8 mg/L for micafungin, and MIC ≥ 1 mg/L for voriconazole were considered resistant (13).

Numerical measurements were summarized as mean ± standard deviation and quarter range, and categorical measurements were summarized as number (percentage). *χ*^2^ test statistics were used to compare categorical measures. IBM SPSS Statistics Version 20.0 (IBM Corp., Armonk, NY, USA) package program was used for statistical data analysis. Statistical significance level in the tests was taken as 0.05 (14). Approval for the study was received from “University Faculty of Medicine” Hospital Clinical Research Ethics Committee (decision number 18, meeting dated 5 December 2023).

## RESULTS

Between 1 January 2010 and 1 August 2023, 471 *C*. *parapsilosis* strains were detected in blood-catheter cultures at University Hospital. Additionally, *C. parapsilosis* was found to be the most common species after *C. albicans*. Of these patients, 200 were included in the study, excluding patients who had *C. parapsilosis* growth again without culture negativity within 1 month and whose data could not be accessed.

**Fig 1:**
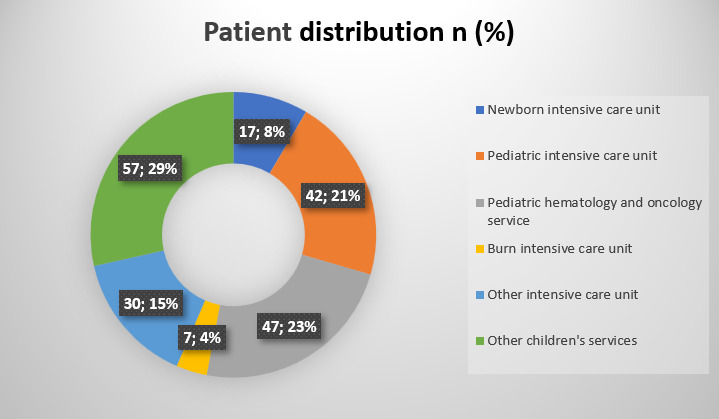
Patient distribution according to clinics, *n* (%).

Of the patients included in the study, 126 (63%) were male. The mean age of the patients was 47.6 months. The distribution of the patients regarding the services in which they were hospitalized is presented in Fig. 1. Most patients were hospitalized in pediatric hematology-oncology and pediatric intensive care units. Eight (4%) patients did not have any chronic disease. Symptoms included fever in 150 (75%), hypothermia in 1 (0.5%), vomiting in 41 (20.5%), diarrhea in 24 (12%), cough in 10 (5%), respiratory distress in 88 (44%), sleepiness in 17 (8.5%), and abdominal pain in 28 (14%). One hundred forty-three of the patients had an intravenous catheter. Catheter-related candidemia was detected in 73 (36.5%) patients, and non-catheter-related candidemia was detected in 127 (63.5%) patients.

**Fig 2:**
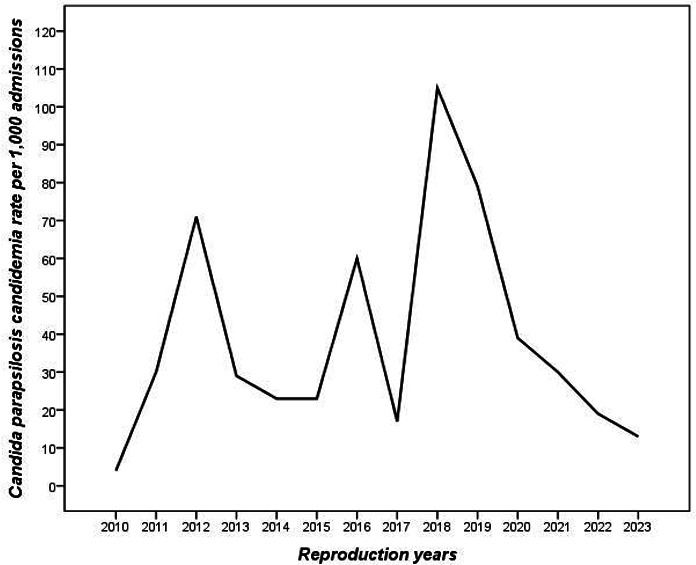
*Candida parapsilosis* candidemia rate per 1,000 admissions.

All patients with candidemia had at least one of the risk factors examined. Clinical and demographic characteristics, underlying chronic diseases, and risk factors for candidemia are presented in Table 1. A significant association was found between malignancy and candidemia-related mortality (*P* = 0.048). In addition, TPN, presence of a central venous catheter, and steroid use were significantly associated with overall mortality (*P* = 0.006, *P* = 0.011, *P* = 0.019, respectively). Nasogastric catheter use did not affect overall mortality, but it was found to affect candidemia-related mortality (*P* = 0.011). The relationship between risk factors, underlying diseases, and overall and candidemia-related mortality is presented in Table 2. The distribution of *C. parapsilosis* strains by years was variable, and the highest rate (14%) was found in 2018. The rate of *C. parapsilosis* candidemia per 1,000 admissions is shown in Fig. 2.

**TABLE 1:** Patients' demographic and clinical features

Features	Number of patients, *n* (%)
Age (months)	
0–1	8 (4.0)
1–12	54 (27.0)
13–60	83 (41.5)
>60	55 (27.5)
Gender	
Female	74 (37)
Male	126 (63)
Candidemia source	
Non-catheter-related candidemia	127 (63.5)
Catheter-related candidemia	73 (36.5)
Presence of intravascular catheter	
Central venous catheter	27 (13.5)
Port	35 (17.5)
Permanent dialysis catheter	1 (0.5)
Catheter tip	10 (5)
Underlying disease	
Non	8 (4.0)
Malignancy	53 (26.5)
Neurometabolic illness^*a*^	38 (19.0)
Congenital heart disease	27 (13.5)
Gastrointestinal system anomaly	25 (12.5)
Premature (<37 gestational week)	15 (7.5)
Central nervous system anomaly	9 (4.5)
Burns patient	7 (3.5)
Chronic liver disease	6 (3.0)
Immunodeficiency	5 (2.5)
Chronic kidney disease	4 (2.0)
Chronic liver disease	2 (1.0)
Down syndrome	1 (0.5)
Risk factor	
Antibiotic use	200 (100.0)
Presence of intravascular catheter	143 (71.5)
History of intensive care stay	111 (55.5)
Nasogastric tube use	107 (53.5)
History of surgical operation	86 (43.0)
Urinary catheter use	82 (41.0)
Mechanical ventilation	81 (40.5)
Hypoalbuminemia	76 (38.0)
H2 blocker use	52 (26.0)
Mucositis	50 (25.0)
Neutropenia	48 (24.0)
Chemotherapy	45 (22.5)
Total parenteral nutrition	35 (17.5)
Steroid treatment	19 (9.5)
Mortality	
General	47 (23.5)
Candidemia	19 (9.5)
Early death	12 (6.0)
Late death	7 (3.5)

^*a*^ The disease is characterized by the inability to synthesize biochemical substances, abnormal accumulation, and damage to brain tissue as a result of mutations that cause biochemical changes in metabolic pathways.

**TABLE 2:** Comparison between alive and dead patients^*a*^^*b*^

Risk factor	Alive (*n* = 153)	Overall mortality (*n* = 47)	*P*	Candidemia-related mortality (*n* = 19)	*P*
Gender					
Female	54 (35.3)	20 (42.6)	0.466	9 (47.4)	0.437
Male	99 (64.7)	27 (57.4)		10 (52.6)	
Age (months)					
0–1	6 (3.9)	2 (4.3)	0.194	2 (10.5)	0.065
1–12	47 (30.7)	7 (14.9)		1 (5.3)	
13–60	61 (39.9)	22 (46.8)		8 (42.1)	
>60	39 (25.5)	16 (34.0)		8 (42.1)	
Primary disease					
Malignancy	36 (23.5)	17 (36.2)	0.126	9 (47.4)	**0.048**
Other	117 (76.5)	30 (63.8)		10 (52.6)	
Premature^*c*^					
None	143 (93.5)	41 (87.5)	0.216	15 (78.9)	0.052
Yes	10 (6.5)	6 (12.8)		4 (21.1)	
Cardiac operation					
None	135 (88.2)	38 (80.9)	0.293	14 (73.7)	0.142
Yes	18 (11.8)	9 (19.1)		5 (26.3)	
Antifungal prophylaxis					
None	136 (88.9)	42 (89.4)	>0.999	15 (78.9)	0.257
Yes	17 (11.1)	5 (10.6)		4 (21.1)	
TPN					
None	133 (86.9)	32 (68.1)	**0.006**	16 (84.2)	0.713
Yes	20 (13.1)	15 (31.9)		3 (15.8)	
Presence of intravascular catheter					
None	51 (33.3)	6 (12.8)	**0.011**	2 (10.5)	0.077
Yes	102 (66.7)	41 (87.2)		17 (89.5)	
Chemotherapy					
None	121 (79.1)	34 (72.3)	0.442	15 (78.9)	>0.999
Yes	32 (20.9)	13 (27.7)		4 (21.1)	
Neutropenia					
None	117 (76.5)	35 (74.5)	0.932	15 (78.9)	>0.999
Yes	36 (23.5)	12 (25.5)		4 (21.1)	
Mechanical ventilator					
None	103 (67.3)	16 (34.0)	**<0.001**	4 (21.1)	**<0.001**
Yes	50 (32.7)	31 (66.0)		15 (78.9)	
Urinary catheter					
None	100 (65.4)	18 (38.3)	**0.002**	4 (21.1)	**<0.001**
Yes	53 (34.6)	29 (61.7)		15 (78.9)	
Nasogastric tube					
None	76 (49.7)	17 (36.2)	0.145	3 (15.8)	**0.011**
Yes	77 (50.3)	30 (63.8)		16 (84.2)	
History of intensive care stay					
None	76 (49.7)	13 (27.7)	**0.013**	4 (21.1)	**0.034**
Yes	77 (50.3)	34 (72.3)		15 (78.9)	
Mucositis					
None	113 (73.9)	37 (78.7)	0.630	16 (84.2)	0.410
Yes	40 (26.1)	10 (21.3)		3 (15.8)	
History of surgical operation					
None	93 (60.8)	21 (44.7)	0.075	9 (47.4)	0.382
Yes	60 (39.2)	26 (55.3)		10 (52.6)	
H2 blocker use					
None	113 (73.9)	35 (74.5)	>0.999	14 (73.7)	>0.999
Yes	40 (26.1)	12 (25.5)		5 (26.3)	
Steroid use					
None	143 (93.5)	38 (80.9)	**0.019**	16 (84.2)	0.160
Yes	10 (6.5)	9 (19.1)		3 (15.8)	

^*a*^ Data are summarized as number (percentage).

^*b*^ *P* < 0.05 was considered significant and are bolded.

^*c*^ Premature: <37 gestational ages week.

Fluconazole prophylaxis was administered before treatment to 22 (11%) patients with newborns weighing <1,500 g and hematological-oncological diseases. No resistance to antifungal agents was detected when the antibiogram susceptibility tests of the patients who received prophylaxis were analyzed.

*C. parapsilosis* growth was accompanied by other microorganisms in 27 (13.5%) patients. These were serious life-threatening microorganisms such as *Acinetobacter baumannii*, *Stenotrophomonas maltophilia*, *Serratia marcescens*, *Enterococcus faecium*, *Staphylococcus hominis*, *Staphylococcus epidermidis*, coagulase negative staphylococci, *Candida famata*, *C. glabrata*, and *C. pellucida*. Microorganism growth in addition to *C. parapsilosis* growth was observed in 3 of 19 patients (15.8%) who died due to candidemia. These were *Acinetobacter baumannii* in one patient, *Stenotrophomonas maltophilia* and *Serratia marcescens* in one patient, and *Candida pellucida* in one patient.

The mean duration of *C. parapsilosis* growth after hospitalization was 41.2 ± 60.3 (3–408) days. The mean duration of *C. parapsilosis* growth after catheterization was 5.3 ± 5 (0–28) days. Nine (4.5%) patients received antifungal amphotericin B lock and systemic therapy. Since the catheters of these patients could not be removed at that time due to their clinical conditions, they were given lock therapy with systemic treatment, and the catheters of all patients were removed as soon as their clinical conditions improved. Of the 73 patients with catheter-associated candidemia, 71 (97.9%) had catheter removal, and 2 (2.7%) did not. It was found that the families of these two patients did not allow port removal. One of the two patients whose port could not be removed died. No ocular or intra-abdominal involvement was detected in *C. parapsilosis* organ involvement. Endocarditis was detected in two (1%) patients.

Of the patients, 194 (97%) had received antifungal treatment for candidemia. Six (3%) patients did not receive antifungal treatment. Of these six patients, two died and four are alive. One hundred sixty-five (82.5%) patients received monotherapy. The most preferred antifungal agents were amphotericin B and fluconazole. The patients' treatment regimens are shown in Table 3. Amphotericin B was most commonly used in the treatment of catheter-associated candidemia. The mean duration of treatment was 19 ± 9.4 (1–64) days.

**TABLE 3:** Summary of patient treatment regimens

Treatment type^*a*^	
Monotherapy	165 (82.5)
Amphotericin B	55 (27.5)
Fluconazole	50 (25.0)
Caspofungin	36 (18.0)
Micafungin	22 (11.0)
Voriconazole	2 (1.0)
Flucytosine	0 (0.0)
Consecutive	18 (9.0)
Combined	9 (4.5)
Consecutive and combine	2 (1.0)
Treatment duration (days)^*b*^	19.4 ± 9.4 19 (10)
Culture negativity duration (days)^*b*^	8.54 ± 8.64 7 (5.5)
Catheter removal duration (days)^*b*^	5.3 ± 5.5 4 (3)

^*a*^ Data are summarized as number (percentage).

^*b*^ Data are summarized as mean ± standard deviation and median (IQR, interquartile range).

The antifungal drug MIC values of *C. parapsilosis* strains are presented in Table 4. The susceptibility-resistance rates of antifungal drugs are also shown in Table 5. Looking at the resistance rates by year, amphotericin B and fluconazole resistance rates were highest in 2021. No correlation was found between antifungal drug resistance and mortality (Table 6).

**TABLE 4:** *Candida parapsilosis* MIC (mg/L) distribution of strains

MIC values (mg/L)												Resistant isolate number
Antifungal agent	≤0.06	≤0.12	≤0.25	≤0.5	≤1	≤2	≥4	≥8	≥16	≥32	≥64	
Amphotericin B	0	0	76	100	8	3^*a*^	1	4	0	0	0	8
Caspofungin	0	2	41	81	34	1	0	3	0	0	0	3
Fluconazole	0	0	7	59	88	27	5	4	4	3	1	12
Voriconazole	0	187	3	5	2^*b*^	0	0	0	0	0	0	2
Micafungin	3	3	3	94	39	4	1	1	0	0	0	1
Flucytosine	0	1	0	2	163	0	0	0	0	0	1	1

^*a*^ For three patients, the amphotericin B MIC value was 2 and they were resistant.

^*b*^ For two patients, the voriconazole MIC value was 1 and they were resistant.

**TABLE 5:** Distribution of *Candida parapsilosis* according to antifungal susceptibility^*b*^

Antifungal agent	Antifungal agent sensitivity			
	Sensitive	Intermediate	Resistant	Unspecified^*a*^
Amphotericin B	184 (92.0)	0 (0.0)	8 (4.0)	8 (4.0)
Caspofungin	159 (79.5)	0 (0.0)	3 (1.5)	38 (19.0)
Fluconazole	181 (90.5)	5 (2.5)	12 (6.0)	2 (1.0)
Voriconazole	190 (95.0)	5 (2.5)	2 (1.0)	3 (1.5)
Micafungin	128 (64.0)	19 (9.5)	1 (0.5)	52 (26.0)
Flucytosine	165 (82.5)	1 (0.5)	1 (0.5)	33 (16.5)

^*a*^ The patient for whom drug resistance testing to the antifungal agent was not performed.

^*b*^ Data are summarized as number (percentage).

**TABLE 6:** Distribution of antifungal susceptibility according to mortality^*a*^^,^^*c*^

		Alive (*n* = 153)	Overall mortality (*n* = 47)	*P^b^*	Candidemia-related mortality (*n* = 19)	*P^b^*
Amphotericin B	S^*d*^	139 (94.6)	45 (100.0)	0.202	18 (100.0)	0.6
	R^*d*^	8 (5.4)	0 (0.0)		0 (0.0)	
	I^*d*^	0 (0.0)	0 (0.0)		0 (0.0)	
Caspofungin	S	123 (97.6)	36 (100.0)	>0.999	14 (100.0)	>0.999
	R	3 (2.4)	0 (0.0)		0 (0.0)	
	I	0 (0.0)	0 (0.0)		0 (0.0)	
Fluconazole	S	141 (92.2)	40 (88.9)	0.12	17 (94.4)	0.241
	R	10 (6.5)	2 (4.4)		0 (0.0)	
	I	2 (1.3)	3 (6.7)		1 (5.6)	
Flucytosine	S	125 (99.2)	40 (97.6)	0.182	15 (100.0)	>0.999
	R	0 (0.0)	1 (2.4)		0 (0.0)	
	I	1 (0.8)	0 (0.0)		0 (0.0)	
Voriconazole	S	146 (96.1)	44 (97.8)	0.731	17 (94.4)	0.702
	R	2 (1.3)	0 (0.0)		0 (0.0)	
	I	4 (2.6)	1 (2.2)		1 (5.6)	
Micafungin	S	104 (85.2)	24 (92.3)	0.61	9 (81.8)	0.891
	R	1 (0.8)	0 (0.0)		0 (0.0)	
	I	17 (13.9)	2 (7.7)		2 (18.2)	

^*a*^ Data are summarized as number (percentage).

^*b*^ *P* < 0.05 was considered significant.

^*c*^ The *χ*^2^ test was performed to compare categorical variables between groups.

^*d*^ S, sensitive; R, resistance; I, intermediate.

The overall mortality rate was 23.5%, and the mortality rate of candidemia was 9.5%. Of the 19 patients who died from candidemia, 14 received monotherapy, 2 received combined treatment, 1 received sequential treatment, and 2 did not receive antifungal treatment. These two patients died before the culture results were obtained.

## DISCUSSION

*C. parapsilosis* is one of the most common invasive fungal infections among non-albicans *Candida* species. In South America and some European countries, *C. parapsilosis* was the most frequently isolated non-albicans *Candida* species in cases of candidemia (15, 16). *C. parapsilosis* has a high reproduction rate, adheres to intravenous devices and prosthetic materials, and has the ability to form biofilms, gastrointestinal colonization, and the possibility of transmission from the colonized hands of healthcare personnel to the patient (17, 18). In a study conducted in the pediatric population, the presence of surgical intervention, *Candida* colonization, chronic lung disease, the presence of indwelling urinary and central venous catheters, and total parenteral nutrition were shown to be risk factors for *C. parapsilosis* candidemia. In this study, mortality rates in patients with *C. albicans*, *C. parapsilosis*, and non-albicans growth were 15.8%, 26%, and 9.5%, respectively (19). In a cohort study, the mortality rate of candidemia was found to be 10%–49% (20). Studies have shown that the most important risk factors for candidemia due to *C. parapsilosis* are the use of central catheters and parenteral nutrition (21, 22). In our study, malignancy, mechanical ventilation, urinary catheter, and nasogastric catheter use were important factors in candidemia mortality, and *C. parapsilosis* mortality rate was 9.5%.

When the distribution of *C. parapsilosis* growth by years was analyzed, it was observed that it was the highest in 2018 and decreased in the following years. We think that this situation is related to the decrease in the admission rates of non-COVID (non-coronavirus disease) diseases to the hospital with the COVID epidemic in the world. In addition, we think that the reason for the low number of cases in 2023 is that our hospital was damaged in our region by the 6 February 2023 earthquake and was partially active at that time.

Accurate identification of the causative agent of fungal infection and determination of the *in vitro* antifungal susceptibility profile make important contributions to the treatment of the patient (23). The emergence of antifungal-resistant strains in non-albicans *Candida* species has again emphasized the importance of *in vitro* antifungal susceptibility testing. Sutcu et al. evaluated 54 pediatric patients with invasive candidemia between 2012 and 2016 at Istanbul Medical Faculty Hospital, a tertiary care facility. *C. parapsilosis* isolates were detected in 13 of them, and amphotericin B and fluconazole resistance was not found in any of them (24). Resistance may not have been detected in this study due to the low number of isolates. In the pediatric study conducted at the tertiary care Izmir Tepecik Hospital, a total of 126 candidemia cases, most commonly *C. parapsilosis*, were detected from 2012 to 2018; within a 7-year period, 33.8% of 71 *C*. *parapsilosis* strains were found to be resistant to fluconazole, which is a high rate (25). In the study conducted by Lotfali et al., in which 120 *C*. *parapsilosis* isolates were examined, they showed that there was 2.5% resistance to fluconazole and 1.7% resistance to amphotericin B (7). In our study, the fluconazole resistance rate was found to be 6%, and the amphotericin B resistance rate was 4%. Blood cultures sent to Kayseri Research Hospital Microbiology Laboratory from various wards and intensive care units between January 2012 and June 2014 were analyzed by Sarıgüzel et al. It was examined retrospectively. Of all the yeast strains, 67 (38.3%) were *C. parapsilosis*. It was determined that fluconazole resistance was 10.4%, and amphotericin B resistance was 8.9% in *C. parapsilosis* strains (26).

In their study, Fidan et al. found the fluconazole resistance rate of *C. parapsilosis* grown in blood cultures of patients hospitalized in the intensive care unit to be 78.6% (27). We think that this high fluconazole resistance rate is related to the very small number of patients in the study, the fact that the patients were intensive care patients, or the hospital’s own resistant flora. In our study, the fluconazole resistance rate of *C. parapsilosis* grown in blood cultures of patients hospitalized in the intensive care unit was 5.7%, which is very low. We think the wide range in this drug resistance rate is due to differences in age group, risk factors, region, and hospital-acquired *C. parapsilosis* strains. In addition, our study found no increase in resistance rate in patients receiving fluconazole prophylaxis. Close follow-up of antifungal susceptibility profiles in each hospital and even between different wards within the hospital is of great importance in empirical treatment selection.

Etiz et al. found that the resistance rate of *C. parapsilosis* to voriconazole was 1%, as in our study. In this study, *C. parapsilosis* flucytosine resistance was not detected (28). In our study, flucytosine drug resistance was found in 0.5%. Pfaller et al. found that the resistance rate to voriconazole in *C. parapsilosis* was 4.3% among 280 non-albicans *Candida* species in the SENTRY antimicrobial surveillance program. In addition, in this study, 1.1% of *C. parapsilosis* species showed intermediate susceptibility to voriconazole (29). Similar results were found in our study.

In invasive candidemia, length of hospital stay, underlying malignancy and immunodeficiency, cardiac failure, use of indwelling urinary catheter and central venous catheter, total parenteral nutrition, and dialysis are factors associated with mortality (19). Knowing local epidemiological data and risk factors in invasive *Candida* infections is very important for empirical treatment.

Catheter removal is of vital importance in catheter-associated bloodstream infections. In our study, one of the two patients whose catheter could not be removed died, and it is seen how important catheter removal is. In a study by Tsai et al., it was shown that only lock treatment in pediatric malignancy patients caused poor results on catheter-associated candidemia mortality (30). Another study suggests that adding an antimicrobial lock solution to systemic antibiotics in pediatric patients may benefit catheter salvage, depending on the etiology (31).

In the treatment of *C. parapsilosis*, fluconazole or amphotericin B is recommended in neutropenic patients, fluconazole can be used in non-neutropenic patients, and if echinocandin is started empirically, it can be continued if there is a clinical and microbiological response; in case of intolerance to other agents or limited benefit, amphotericin B treatment should be given (21).

As a result, it appears that the risk of *C. parapsilosis* candidemia is high in patients who are monitored using mechanical ventilation devices and have nasogastric and urinary catheters and central catheters. It should be considered that response to treatment will be difficult, especially in patients with malignancy and these risk factors.

Considering that antifungal drugs develop resistance against *C. parapsilosis*, we think that performing antifungal sensitivity tests in every center will guide empirical treatment and that empirical antifungal use policies in clinics should be re-evaluated in the light of this information. The retrospective nature of our study and the lack of isolate subtyping of *C. parapsilosis* complex members created limitations in our study. We believe that for treating catheter-related candidemia, the catheter should be removed first; if it cannot be removed, lock therapy should be administered along with systemic therapy and the catheter should be removed as soon as possible.
